# Cardiovascular risk factor burden and adverse pregnancy outcomes in women with cardiovascular disease^☆^

**DOI:** 10.1016/j.ahjo.2025.100611

**Published:** 2025-09-11

**Authors:** Valerie C. Nemov, Alden Dunham, Claudio Schenone Giugni, Viviana De Assis, Emily Coughlin, Mary Ashley Cain, Judette M. Louis, Daniela R. Crousillat

**Affiliations:** a University of South Florida, Morsani College of Medicine; 560 Channelside Drive, Tampa, FL, 33602, USA; b Division of Maternal-Fetal Medicine, Department of Obstetrics and Gynecology, University of South Florida, 2 Tampa General Circle 6th Floor, Tampa, FL, 33606, USA; c Division of Cardiovascular Sciences, Department of Internal Medicine, University of South Florida, 2 Tampa General Circle 5th Floor, Suite 5072, Tampa, FL, 33606, USA

**Keywords:** Cardiovascular disease, Pregnancy, Adverse pregnancy outcomes, Hypertensive disorder of pregnancy, Cardio-obstetrics

## Abstract

**Introduction:**

We investigated associations between pre-conception cardiovascular risk factor burden and adverse pregnancy outcomes (APOs) in women with cardiovascular disease (CVD).

**Methods:**

We created a patient registry from our cardio-obstetrics program. APOs were defined as intrauterine growth restriction (IUGR), hypertensive disorder of pregnancy (HDP), and pre-term birth.

**Results:**

Analysis included 63 women. 42 (66.7 %) experienced no APOs, while 21 (33.3 %) did; 18 (28.6 %), 3 (4.8 %), and 12 (19.0 %) developed an HDP, IUGR, or delivered pre-term, respectively. Pre-conception risk burden was not a significant predictor of APO development (*p* *=* 0.139). However, patients with pre-term delivery had a significantly higher number of risk factors (*p* < 0.001), as did patients with chronic hypertension with superimposed HDP (*p* < 0.001).

**Discussion:**

Women delivering pre-term have higher risk factor burdens. Since pre-term birth is associated with future CVD in women independent of cause, optimization of preconception cardiovascular health could help mitigate these risks in an already vulnerable population.

## 1 Introduction

Cardiovascular disease (CVD) affects 1–4 % of pregnancies in the United States and is the primary contributor to morbidity and mortality in pregnancy and post-partum [1,2]. While adverse pregnancy outcomes (APOs) are associated with increased risk of future maternal CVD, the factors predicting these pregnancy complications are less well-studied. We report preliminary data from our retrospective cardio-obstetrics registry on the association between pre-conception cardiovascular risk factor burden and APO development and contribute cardio-obstetrics patient data to the literature.

## 2 Methods

We completed a retrospective cohort study of all patients evaluated at a single academic center's cardio-obstetrics program from October 2021 through November 2022. For analysis purposes, we excluded patients presenting preconception who did not become pregnant and deliver in the study period, those with incomplete data, and patients without CVD. Patients without CVD were those referred to the program for new cardiac symptoms in pregnancy (such as shortness of breath, palpitations, edema, orthostatic hypotension, and presyncope) who had a negative cardiac evaluation and work-up (i.e. sinus tachycardia, low (<5 %) burden PAC/PVCs on ambulatory heart monitor, etc.). Patients with CVD were defined as structural or arrhythmogenic heart disease (see Table 1 for stratification).

**Table 1:** Indications for cardio-obstetrics care among all included patients.

Indication for cardio-obstetrics care	Number of patients (%)
Arrhythmia	10 (15.9)
Valvular or structural heart disease	21 (33.3)
Aortopathy	9 (14.3)
Cardiomyopathy	18 (28.6)
Congenital heart disease	4 (6.3)
Ischemic heart disease	9 (14.3)
Embolic or hypercoagulable disease	5 (7.9)
Pulmonary hypertension	3 (4.8)
Other CVD	2 (3.2)

While the majority of included patients were referred to the cardio-obstetrics program after conception, their records were retrospectively reviewed from the first antenatal appointment of their current pregnancy through six weeks post-partum. Patient charts were queried for demographics, clinical characteristics, pre-conception cardiovascular risk factors, and maternal-fetal outcomes. Risk factors included BMI >25 kg/m^2^, chronic hypertension (cHTN), hyperlipidemia, pre-existing diabetes, tobacco use, sleep apnea, chronic kidney disease, autoimmune disease, advanced maternal age, and history of a prior APO.

The primary outcome was the risk of experiencing an APO based on pre-conception cardiovascular risk factor burden. APOs were defined as fetal growth restriction (FGR), pre-term birth (delivery at <37 weeks), and hypertensive disorder of pregnancy (HDP). We defined HDP as gestational hypertension, preeclampsia, eclampsia, HELLP syndrome, and cHTN with superimposed preeclampsia.

For comparisons of categorical variables, chi-square or Fisher's exact test were applied. Mann-Whitney *U* test was used to assess continuous variables including number of risk factors. The significance threshold was *p* < 0.05. This study was approved by the University of South Florida IRB.

## 3 Results

A total of 112 patients received care in the cardio-obstetrics program during the study period. Sixty-three patients met inclusion criteria; their indications for care are detailed in Table 1. Over the follow-up period, 42 (66.7 %) women did not experience any APO, while 21 (33.3 %) did; 18 (28.6 %) women developed an HDP, 3 (4.8 %) women experienced FGR (all confirmed SGA at birth), and 12 (19.0 %) women delivered pre-term.

Analysis of demographic data showed the only significant differences between those who experienced an APO and those who did not was their gestational age at delivery and infant's birthweight (Table 2); these were both expected given that pre-term birth was included as an APO. However, we conducted a sensitivity analysis excluding pre-term birth as an APO, and the results were not significantly affected. No individual cardiovascular risk factor was significantly associated with the development of an APO.

**Table 2:** Demographics and primary clinical outcome organized by presence or absence of an APO.

	Total (*N* = 63)	No APO (*N* = 42)	Any APO (*N* = 21)	*p*-Value
Race, self-reported^a^				0.622
White	33 (52.3)	21 (50.0)	12 (57.1)	
Black	18 (28.6)	13 (31.0)	5 (23.8)	
Asian	1 (1.6)	0 (0.0)	1 (4.8)	
Other	9 (14.3)	6 (14.3)	3 (14.3)	
More than one race	2 (3.2)	2 (4.8)	0 (0.0)	
Ethnicity, self-reported^a^				0.682
Hispanic/Latino	16 (25.4)	10 (23.8)	6 (28.6)	
Not Hispanic/Latino	47 (74.6)	32 (76.2)	15 (71.4)	
Insurance status^a^				0.469
Medicaid	26 (41.3)	16 (38.1)	10 (47.6)	
Private/Commercial	37 (58.7)	26 (61.9)	11 (52.4)	
Age (years)^b^	32.2 ± 5.8	31.7 ± 5.5	33.2 ± 6.4	0.332
Gravidity^b^	2.8 ± 1.5	2.8 ± 1.5	2.7 ± 1.6	0.889
Parity^b^	1.1 ± 1.0	1.1 ± 1.1	1.3 ± 1.0	0.232
Gestational age at first visit (weeks)^b^	25.7 ± 7.9	25.4 ± 8.3	26.7 ± 6.9	0.686
Gestational age at delivery (weeks)^b^	37.6 ± 2.4	38.6 ± 1.0	35.7 ± 3.1	<0.001
Birthweight (g)^b^	2988.5 ± 703.1	3226.3 ± 457.8	2524.2 ± 864.0	0.003
Risk factors^a^				
BMI > 25 kg/m^2^	42 (66.7)	28 (66.7)	14 (66.7)	1.000
cHTN	16 (25.4)	8 (19.0)	8 (38.1)	0.102
Hyperlipidemia	1 (1.6)	1 (2.4)	0 (0.0)	1.000
Pre-existing diabetes	5 (7.9)	4 (9.5)	1 (4.8)	0.657
Tobacco use	9 (14.3)	6 (14.3)	3 (14.3)	1.000
Sleep apnea	1 (1.6)	0 (0.0)	1 (4.8)	0.333
Chronic kidney disease	2 (3.2)	0 (0.0)	2 (9.5)	0.108
Autoimmune disease	9 (14.3)	6 (14.3)	3 (14.3)	1.000
Advanced maternal age	19 (30.2)	11 (26.2)	8 (38.1)	0.332
Prior APO	19 (30.2)	11 (26.2)	8 (38.1)	0.332
Primary outcome^b^				
Total number of risk factors	1.95 ± 1.28	1.79 ± 1.22	2.29 ± 1.35	0.139

a Reported at number of patients (%).

b Reported as mean ± standard deviation.

Analysis of risk factor burden showed that the number of risk factors a woman had pre-conception was not a statistically significant predictor of APO development (*p* *=* 0.139). Patients who experienced pre-term birth had a significantly higher number of risk factors (*p* < 0.001; 3.08 ± 1.00 pre-term, 1.69 ± 1.19 not pre-term) than those without preterm birth. Patients with cHTN with superimposed preeclampsia also had a significantly higher number of risk factors (*p* < 0.001; 3.43 ± 0.79 with, 1.77 ± 1.21 without).

## 4 Discussion

In our analysis, cardiovascular risk factor burden was not associated with APO development in pregnant women with CVD. While we found that women who developed cHTN with superimposed preeclampsia had a significantly higher number of risk factors than women who did not, these patients had cHTN at baseline and therefore automatically had an additional risk factor. Additionally, women who delivered pre-term had a significantly higher number of risk factors than women who delivered at term. This likely occurred because women who enter pregnancy with more CVD risk factors are likely to have pregnancy complications that may necessitate early delivery, including a hypertensive disorder of pregnancy. Notably, 9 (75.0 %) women who experienced pre-term delivery also had an HDP; only 9 (17.6 %) of the women who did not deliver pre-term had an HDP. We did not differentiate between women with spontaneous versus medically induced pre-term deliveries. Since pre-term birth is associated with future CVD morbidity and mortality in women independent of its cause [1,3], optimization of preconception cardiovascular health could mitigate these risks in an already vulnerable high-risk population.

In selecting pre-conception risk factors for the development of an APO, we included chronic kidney disease (CKD). In a large systematic review and meta-analysis, pre-pregnancy chronic kidney disease was associated with an increased risk of pre-eclampsia, pre-term birth, and small for gestational age (SGA) babies [4]. In our study population, two patients had CKD: one secondary to right renal atrophy with left renal artery stenosis and one with unspecified antepartum renal disease. We also included autoimmune disease as a risk factor, as a range of inflammatory and systemic rheumatic diseases have been closely associated with increased risk of APOs [5,6]. In our study, nine patients had autoimmune disease spread across rheumatic disease, systemic lupus erythematosus, and Hashimoto's disease.

The optimal time for women with known CVD to receive cardiovascular evaluation is preconception, as adverse cardiac events in this population have been shown to be largely preventable with proper and early multidisciplinary cardio-obstetrics care [1,7]. Referrals to our cardio-obstetrics clinic are made by general OB/GYN practitioners within our institution as well as maternal fetal medicine physicians either pre-conception, during pregnancy, or postpartum. Referral criteria includes pre-existing cardiac disease, suspected cardiac disease in pregnancy, new cardiac symptoms in pregnancy, the development of postpartum cardiac conditions (such as cardiomyopathy), or a history of high-risk disease (such as pulmonary embolism). Patients are seen in our institution's cardio-obstetrics clinic, where cardiac evaluation, workup, and follow-up is completed by the program's cardiologist and maternal fetal medicine specialists as needed. Obstetric/cardiac anesthesia and adult and/or neonatal intensive care unit teams are involved on an individualized basis. The gestational age at time of referral to our cardio-obstetrics program was documented for all patients to assess referral patterns. Among all included patients, the average gestational age at the time of initial cardio-obstetrics evaluation was 25.7 weeks gestation. As most women presenting to our program had established CVD prior to pregnancy, future efforts should include promoting engagement within the program earlier—ideally pre-conception.

Limitations of this study are its small sample size and retrospective nature. Additionally, patients were identified based on appointments at the cardio-obstetrics clinic. Therefore, there is a population of pregnant women with CVD who met this study's criteria yet were not included due to referral patterns. The study cohort has a great deal of heterogeneity in the presenting cardiovascular conditions, and each of their unique contributions need to be clarified in future studies. Lastly, outcomes may not be generalizable to all populations of pregnant women with CVD, as access to expert cardio-obstetrics clinics, such as the one at our institution, is limited and may lead to treatment bias.

Prior studies have suggested a link between CVD risk factor burden and risk of adverse pregnancy outcomes. Based on an analysis of National Center for Health Statistics data, there is a consistent and graded association between worse pre-pregnancy cardiovascular risk factor burden and APOs (preterm birth, SGA birth, and fetal death) [8]. However, the link between preconception CVD risk factors and risk of APOs has not been previously reported among a population of women with existent cardiovascular disease as in our study. Our preliminary findings show that cardiovascular risk factor burden is not a statistically significant predictor of APO development in pregnant women with CVD. Larger studies are needed to further investigate the impact of pre-conception risk factors on outcomes in pregnant women with CVD and to expand our knowledge of the impact cardio-obstetrics programs have on their care trajectories.

## Funding disclosures/disclaimers

None to disclose.

## CRediT authorship contribution statement

**Valerie C. Nemov:** Writing – review & editing, Visualization, Conceptualization, Methodology, Writing – original draft, Investigation. **Alden Dunham:** Investigation, Writing – review & editing. **Claudio Schenone Giugni:** Writing – review & editing, Conceptualization, Methodology. **Viviana De Assis:** Writing – review & editing, Methodology, Conceptualization. **Emily Coughlin:** Writing – original draft, Formal analysis. **Mary Ashley Cain:** Writing – original draft, Project administration, Supervision, Conceptualization, Writing – review & editing, Methodology. **Judette M. Louis:** Methodology, Writing – review & editing, Resources, Supervision. **Daniela R. Crousillat:** Supervision, Project administration, Data curation, Writing – review & editing, Resources, Investigation, Writing – original draft, Methodology, Conceptualization.

## Ethical statement

All procedures were performed in compliance with relevant laws and institutional guidelines and have been approved by the appropriate institutional committee. Patients' privacy was observed and protected during this study.

Ethical approvals obtained:

University of South Florida IRB
Date of approval: 3/15/2023
Reference number: STUDY005033

## Declaration of competing interest

The authors declare that they have no known competing financial interests or personal relationships that could have appeared to influence the work reported in this paper.
